# Dried tea residue can alter the blood metabolism and the composition and functionality of the intestinal microbiota in Hu sheep

**DOI:** 10.3389/fmicb.2023.1289743

**Published:** 2023-11-03

**Authors:** Liangyong Guo, Shiqiang Yu, Fang Cao, Kaizhi Zheng, Manman Li, Zhenying Peng, Xingyun Shi, Liping Liu

**Affiliations:** ^1^Huzhou Key Laboratory of Innovation and Application of Agricultural Germplasm Resources, Huzhou Academy of Agricultural Sciences, Huzhou, China; ^2^Laboratory of Gastrointestinal Microbiology, Jiangsu Key Laboratory of Gastrointestinal Nutrition and Animal Health, College of Animal Science and Technology, Nanjing Agricultural University, Nanjing, China; ^3^College of Life Science, Huzhou Teachers College, Huzhou, China; ^4^Institute of Animal Husbandry and Veterinary, Zhejiang Academy of Agricultural Sciences, Hangzhou, China; ^5^Key Laboratory of Animal Physiology and Biochemistry, College of Veterinary Medicine, Nanjing Agricultural University, Nanjing, China; ^6^Beijing Jingmi Water Diversion Management Office, Beijing, China

**Keywords:** dried tea residue, Hu sheep, serum, immune, antioxidant, intestinal microorganisms

## Abstract

Ruminant animals face multiple challenges during the rearing process, including immune disorders and oxidative stress. Green tea by-products have gained widespread attention for their significant immunomodulatory and antioxidant effects, leading to their application in livestock production. In this study, we investigated the effects of Dried Tea Residue (DTR) as a feed additive on the growth performance, blood biochemical indicators, and hindgut microbial structure and function of Hu sheep. Sixteen Hu sheep were randomly divided into two groups and fed with 0 and 100 g/d of DTR, respectively. Data were recorded over a 56-day feeding period. Compared to the control group, there were no significant changes in the production performance of Hu sheep fed with DTR. However, the sheep fed with DTR showed a significant increase in IgA (*p* < 0.001), IgG (*p* = 0.005), IgM (*p* = 0.003), T-SOD (*p* = 0.013), GSH-Px (*p* = 0.005), and CAT (*p* < 0.001) in the blood, along with a significant decrease in albumin (*p* = 0.019), high density lipoprotein (*p* = 0.050), and triglyceride (*p* = 0.021). DTR supplementation enhanced the fiber digestion ability of hindgut microbiota, optimized the microbial community structure, and increased the abundance of carbohydrate-digesting enzymes. Therefore, DTR can be used as a natural feed additive in ruminant animal production to enhance their immune and antioxidant capabilities, thereby improving the health status of ruminant animals.

## Introduction

Ruminant animals, while having high production capacity, often face issues such as oxidative stress and metabolic disorders, primarily due to the high metabolic load and decreased immune adaptability (Oh et al., 2017; Gonzalez-Rivas et al., 2020). Natural plants have been recognized as potential remedies to improve animal health and maintain metabolic homeostasis (Besharati and Taghizadeh, 2009; Ramdani et al., 2023). Due to increasing concerns regarding the side effects of antibiotic drugs, their usage has been widely scrutinized and prohibited as feed additives (Tang et al., 2017). Tea, a widely consumed plant, contains a significant amount of active compounds such as polyphenols. These compounds possess properties that can promote human and animal health (Yan et al., 2020). In ruminant animals, feeding diets supplemented with polyphenol-rich ingredients has shown significant biological effects. These effects include improved animal productivity, enhanced quality of livestock products, increased immune competence, and reduced rumen methane emissions, among others (Jiménez-Ocampo et al., 2019; Ku-Vera et al., 2020; Suresh et al., 2023). Therefore, plants rich in polyphenols and other active compounds have been used in the production of ruminants (Maghsoud et al., 2008; Ramdani et al., 2013). Tea, for example, generates a significant amount of byproducts during processing, which contain abundant active ingredients such as polyphenols, polysaccharides, and catechins. As a result, tea extracts and other derivatives are being increasingly applied in the pharmaceutical and food industries (Duarah et al., 2023). Polyphenols, such as epicatechin and epigallocatechin gallate, are major components found in tea. They exhibit various biological activities, including antioxidant, anti-inflammatory, and anti-stress properties (Teixeira Oliveira et al., 2023).

There have been previous reports indicating that tea and its byproducts can serve as a source of protein, fiber, secondary metabolites, and minerals in ruminant diets. They can be used as natural feed additives for ruminant animals and have the advantage of reducing methane emissions and minimizing resource wastage (Zebeli and Ametaj, 2009; Sezmis et al., 2023). In the study conducted by Chowdhury et al. (2022), it was reported that dried green tea byproducts can improve protein digestibility in goats and increase plasma glucose concentrations. Furthermore, the abundant polyphenols found in green tea can reduce oxidative stress in ruminant animals. For example, it significantly lowers somatic cell counts in periparturient cows and decreases concentrations of triglycerides, reactive oxygen species, malondialdehyde, and hydrogen peroxide (Ma et al., 2021). It also increases the concentrations of glutathione peroxidase, superoxide dismutase, and total antioxidant capacity. Additionally, it upregulates the concentrations of IL-6 and IL-10 in plasma while downregulating the concentrations of TNF-α, IL-1β, IL-2, IL-8, and IFN-γ (Ma et al., 2021). These effects help reduce oxidative stress in cows and improve their lactation performance and overall health status. The polyphenols present in green tea can also inhibit the expression of TGF-β1 in bovine mammary glands, thereby reducing the phosphorylation of p38 and JNK. This leads to a significant decrease in the expression of inflammatory cytokines IL-1β, IL-6, and TNF-α (Xu et al., 2022). Additionally, green tea polyphenols can alleviate oxidative stress, inflammation, and cell apoptosis in bovine mammary epithelial cells induced by hydrogen peroxide. This effect is achieved through the activation of the ERK1/2-NFE2L2-HMOX1 pathway (Ma et al., 2022). Indeed, green tea compounds can also alter the fermentation in the rumen of ruminant animals and the composition of their intestinal microbiota (Qiu et al., 2021; Gao et al., 2022). These reports indicate that green tea can be utilized as an antioxidant additive and a microbial modulator in ruminant animals production.

The active effects exerted by tea are mainly determined by its major constituents and their metabolism within ruminant animals. Tea polyphenols and EGCG are the most significant active components. For instance, tea polyphenols can have beneficial effects on the cellular redox balance of animals, reducing oxidative stress-related damage and potentially serving as antioxidants in animal antioxidant defense against oxidative stress (Ma et al., 2018; Xu et al., 2021). On the other hand, it is documented that the interaction between the gastrointestinal tract of ruminant animals and polyphenols plays a crucial role in mediating the promotion of host health by plant-derived polyphenols. For instance, these interactions can influence the structure and community of the gastrointestinal microbiota, promoting beneficial bacteria and inhibiting harmful bacteria (Yu et al., 2023). The gut microbiota of ruminant animals can further metabolize active substances such as polyphenols, thereby enhancing their bioavailability and utilization (Bhat et al., 1998). Consequently, the active substances produced through these metabolisms may improve oxidative stress and inflammatory responses in ruminant animals, regulate gastrointestinal function, and ultimately enhance microbial growth and the overall health status of the animals.

The immune status and gut health of sheep significantly influence their growth performance and milk production capacity. Previous studies have indicated that tea leaves and tea waste have the ability to regulate rumen fermentation, reduce methane emissions, and improve immune status in animals (Qiu et al., 2021; Chowdhury et al., 2022). However, there is limited research on the effects of tea-related substances on blood metabolism and the composition and functionality of the hindgut microbiota in sheep. Gaining a better understanding of the microbial community and their functional responses to tea components can help develop mechanisms for manipulating the gut microbiota using natural plant compounds, thereby improving the growth status and health of sheep. We hypothesize that adding dried tea waste to the diet can improve the immune and antioxidant status of sheep by modulating the gut microbiota. We aim to study the effects of tea waste on sheep’s gut functionality using metagenomics and other related methods, evaluate its impact on blood antioxidant and immune indicators, and uncover the mechanisms by which tea waste influences the sheep’s hindgut and improves their overall health status.

## Materials and methods

### Source of dried tea residue

The dried tea residue we selected is derived from the by-products remaining from the production process of Anji white tea in Anji County. The main components of this by-product are tea polyphenols (18.10%), L-theanine (4.09%), catechin (14.75%), and epigallocatechin gallate (EGCG) ester of gallic acid (13.00%).

### Animals and treatments

The 16 male Hu sheep weighing 29.80 ± 0.91 kg at 3 months of age were randomly divided into a control group and a treatment group, with eight Hu sheep in each group. The control group was fed a basal diet (CON), while the treatment group was fed 100 g/d of dried tea residue (DTR). The dosage of DTR was determined based on the results of an *in vitro* experiment (unpublished). The basal diet (Supplementary Table 1) was a complete mixed ration with a concentrate-to-roughage ratio of 7: 3, meeting the requirements of the Chinese Sheep Feeding Standards (NY/T816-2004). The Hu sheep in the experiment were individually housed in a pen and were fed twice a day (at 8:00 and 17:00 h). They had free access to feed and water, and daily feed intake was recorded. Initial and final body weights were recorded. The entire experiment lasted for 56 days, including a 14-day adaptation period and the formal experimental period was 42-day. On the last day of the experiment, 2 h before morning feeding, blood samples were collected via jugular vein puncture, and feces were collected.

### Serum sampling and analysis

After collecting the blood samples from the jugular veins of the Hu sheep using non-anticoagulant vacuum tubes before morning feeding, the samples were centrifuged at 3,000 × *g* for 10 min at 4°C to collect the serum. Subsequently, the serum was frozen at −80°C until analysis. The concentrations of total superoxide dismutase (T-SOD), glutathione peroxidase (GSH-Px), total antioxidant capacity (T-AOC), catalase (CAT), malondialdehyde (MDA), total protein content (TP), albumin (ALB), high density lipoprotein (HDL), low density lipoprotein (LDL), glutamic pyruvic transaminase (GPT), glutamic-oxalacetic transaminase (GOT), nonesterified fatty acid (NEFA), triglyceride (TG) and total cholesterol (TCH) were determined using the appropriate commercial assay kits (Nanjing Jiancheng Bioengineering Institute, Nanjing, China) and microplate reader (Multiskan FC; Thermo Fisher Scientific, Waltham, MA, USA) analyzer. And were analyzed using commercial ELISA assay kits (Nanjing Jiancheng Bioengineering Institute, Nanjing, China), following the instructions provided by the supplier. All ELISA data were recorded using a microplate reader (Multiskan FC; Thermo Fisher Scientific, Waltham, MA, USA).

### Hindgut microbial analysis by metagenomic sequencing

Microbial DNA was extracted from feces samples. The concentrations of Immunoglobulin A (IgA), Immunoglobulin G (IgG), and Immunoglobulin M (IgM) using the E.Z.N.A.® stool DNA Kit (Omega Bio-tek, Norcross, GA, U.S.) according to manufacturer’s protocols. Metagenomic shotgun sequencing libraries were constructed and sequenced at Shanghai Biozeron Biological Technology Co. Ltd. In briefly, for each sample, 1 μg of genomic DNA was sheared by Covaris S220 Focused-ultrasonicator (Woburn, MA USA) and sequencing libraries were prepared with a fragment length of approximately 450 bp. All samples were sequenced in the Illumina NovaSeq 6000 instrument with pair-end 150 bp (PE150) mode.

The quality control of each dataset was performed using Fastp (version 0.20.0, https://github.com/OpenGene/fastp). This involved trimming the 3′-end and 5′-end of reads, cutting low-quality bases (quality scores <20), and removing short reads (<50 bp) and “N” records. The reads were then aligned to the host genome^1^ using BWA (version 0.7.17, http://bio-bwa.sourceforge.net/) to filter out host DNA. The filtered reads were *de novo* assembled for each sample using Megahit (Li et al., 2015; version 1.1.2, https://github.com/voutcn/megahit). Prodigal^2^ was employed to predict open reading frames (ORFs) from the assembled contigs with a length > 100 bp. The assembled contigs were then pooled, and non-redundant sequences were generated based on identical contigs using CD-HIT (Fu et al., 2012; version v4.6.1, http://weizhongli-lab.org/cd-hit/) with 90% identity. To determine the gene abundance information in each corresponding sample, the high-quality reads of each sample were compared with the non-redundant gene set using SOAPaligner (Li et al., 2009; http://soap.genomics.org.cn/; default parameters: 95% identity).

The non-redundant gene set was subjected to a comparison with the NR database using DIAMOND (Buchfink et al., 2015) software, with the comparison type set to BLASTP. Species annotations were obtained from the taxonomic information database corresponding to the NR database.^3^ The abundance of species in each samples were counted at each taxonomic level, including domain, family, genus, and species, to construct an abundance profile at the corresponding taxonomic level. Principal Coordinate Analysis (PCoA) based on the Bray-Curtis similarity matrix was conducted at the species level. Contigs were annotated using DIAMOND against the KEGG database (Kanehisa, 2000; Kyoto Encyclopedia of Genes and Genomes, http://www.genome.jp/kegg/) with an E-value of 1e-5. Furthermore, the non-redundant gene set was compared with the CAZy database^4^ using the corresponding tool hmmscan from the CAZy database to obtain annotation information of carbohydrate-active enzymes corresponding to the genes. The abundances of KEGG Orthology (KO), pathway, KEGG enzyme, and CAZymes were normalized into counts per million reads (cpm) for further analysis. For downstream analysis, at least 50% of the animals in each group were used. KEGG modules, pathways, KEGG enzymes, and CAZymes with cpm > 5 were considered for the analysis.

The complete set of assembled and filtered raw sequence data has been submitted to the NCBI Sequence Read Archive, and it is now available under bioproject PRJNA1002066.

### Statistical analysis

The data for growth performance and blood parameters were analyzed using SPSS 21.0 software (SPSS Inc., Chicago, IL, United States). After testing for normal distribution, a double-tailed *t*-tests was employed for analysis. The *p*-value ≤ 0.05 was considered as indicating a significant difference, while *p*-value > 0.05 indicated no significant difference.

## Results

### Growth performance

Table 1 reports the variations in the productive performance indicators of Hu sheep in the experiment. There were no significant differences observed in the initial weight and terminal weight between the CON and DTR groups during the course of the study (*p* > 0.05). Additionally, no significant differences were found in average daily feed intake, average daily gain, and Feed/Gain in this research (*p* > 0.05).

**Table 1:** Effects of DTR supplementation on growth performance of Hu sheep.

Item	Treatments		SEM	*p*-value
	CON	DTR		
Initial weight, kg	30.19	29.41	0.23	0.092
Terminal weight, kg	40.68	40.08	0.78	0.708
Average daily feed intake, g	1447.48	1514.57	69.11	0.644
Average daily gain, g	205.88	209.19	14.26	0.912
Feed/Gain	7.26	7.38	0.22	0.808

### Serum index

Table 2 describes the differences in biochemical indicators in the serum of Hu sheep after the addition of DTR. It can be observed that the concentrations of IgA (*p* < 0.001), IgG (*p* = 005), IgM (*p* = 0.003), T-SOD (*p* = 0.013), GSH-Px (*p* = 0.005), and CAT (*p* < 0.001) in the serum of Hu sheep were significantly higher in the DTR group compared to the CON group, indicating that DTR has the function of enhancing the immunity and antioxidation of Hu sheep. Additionally, the concentrations of ALB (*p* = 0.019), HDL (*p* = 0.050), and TG (*p* = 0.021) in the serum were significantly lower in the DTR group compared to the CON group after DTR supplementation. After the addition of DTR, there were no significant changes observed in the indicators T-AOC, MDA, TP, LDL, GPT, GOT, NEFA, and TCH (*p* > 0.05).

**Table 2:** Effect of TEA on the serum index of sheep.

Item	Treatments		SEM	*p*-value
	CON	DTR		
IgA, μg/ml	1117.83^b^	1566.26^a^	293.24	<0.001
IgG, mg/ml	4.02^b^	4.99^a^	0.75	0.005
IgM, μg/ml	142.57^b^	296.4^a^	40.33	0.003
T-SOD, U/ml	59.31^b^	76.18^a^	3.61	0.013
GSH-Px, U/ml	111.51^b^	128.75^a^	3.34	0.005
T-AOC, U/ml	3.24	3.62	0.14	0.190
CAT, U/ml	2.68^b^	2.92^a^	0.04	<0.001
MDA, nmol/ml	3.23	3.29	0.09	0.757
TP, g/L	68.49	69.50	0.49	0.317
ALB, g/L	27.70^a^	23.90^b^	0.85	0.019
HDL, mmol/L	0.45^a^	0.30^b^	0.04	0.050
LDL, mmol/L	1.00	1.01	0.02	0.889
GPT, U/L	29.65	29.37	1.77	0.941
GOT, U/L	126.77	127.27	0.14	0.072
NEFA, μmol/L	138.50	142.27	5.46	0.743
TG, mmol/L	0.47^a^	0.37^b^	0.02	0.021
TCH, mmol/L	1.26	1.10	0.09	0.414

T-SOD, total superoxide dismutase; GSH-Px, glutathione peroxidase; T-AOC, total antioxidant capacity; CAT, catalase; MDA, malondialdehyde; TP, total protein content; ALB, Albumin; HDL, high density lipoprotein; LDL, low density lipoprotein; GPT, glutamic pyruvic transaminase; GOT, glutamic-oxalacetic transaminase; NEFA, nonesterified fatty acid; TG, triglyceride; TCH, total cholesterol. Values in the same row with the same or no small letter superscripts mean no significant difference (*p* > 0.05), while with different letter superscripts mean significant difference (*p* ≤ 0.05).

### Metagenome profiling

**Figure 1:**
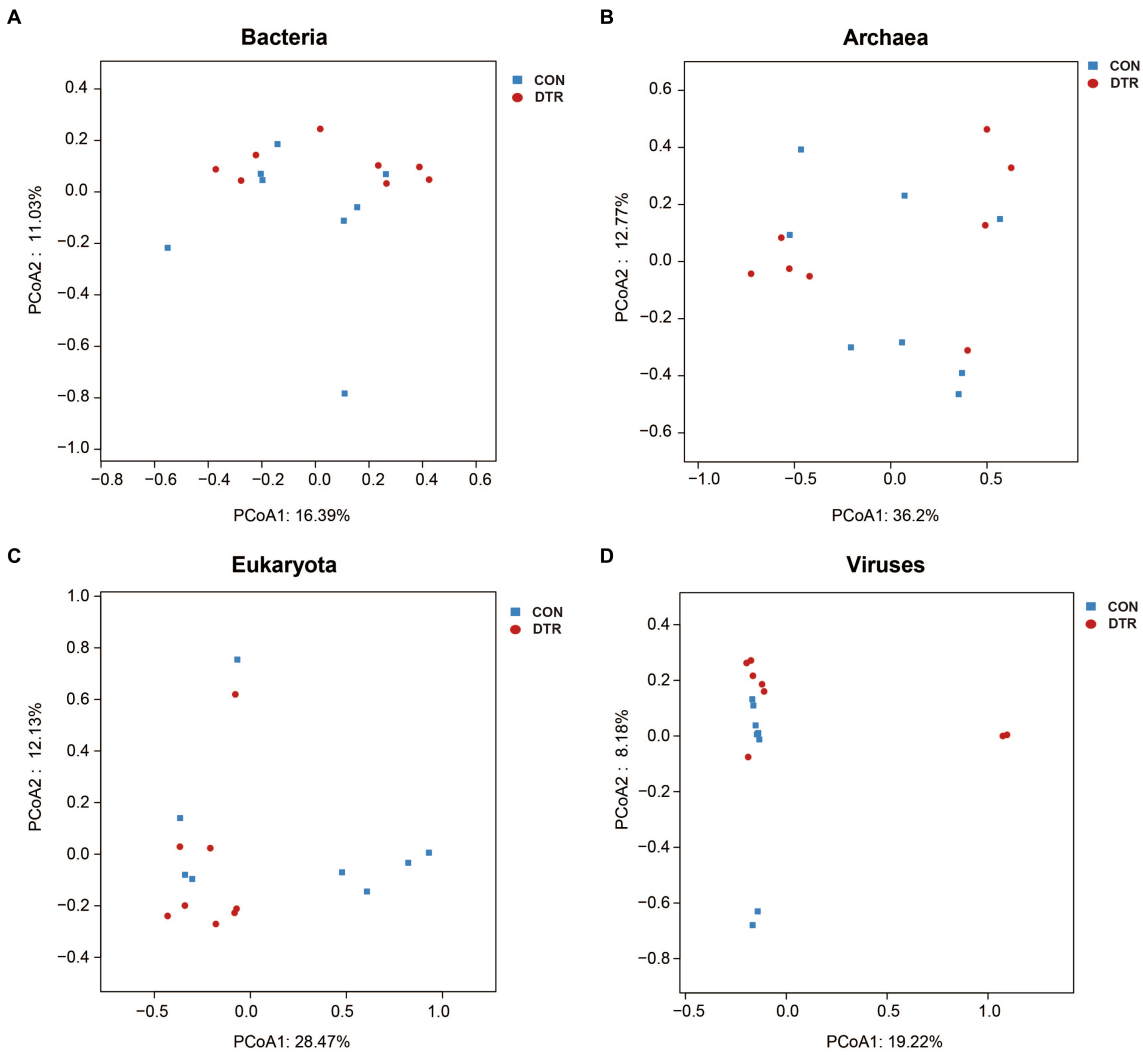
Hindgut microbial structure analysis at the domain level. The compositional profiles of bacteria **(A)**, eukaryota **(B)**, archaea **(C)**, and viruses **(D)** based on PCoA.

**Figure 2:**
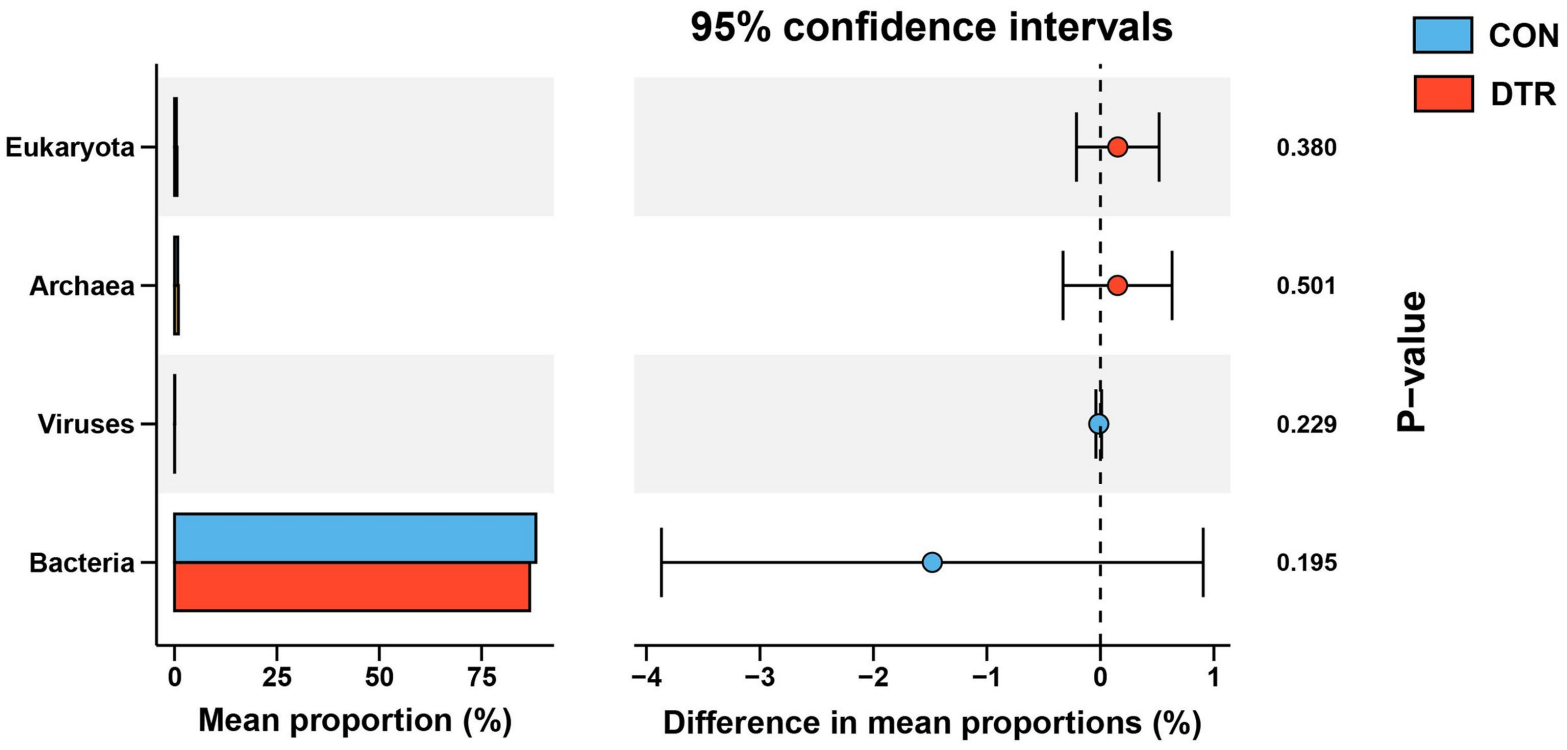
Comparison of microbial domains between CON and DTR sheep. Significantly different domains were tested by Wilcoxon rank-sum test.

Metagenomic sequencing of the total DNA from 16 rumen fluid samples generated a total of 1,866,654,428 reads, with an average of 116,665,901 ± 3,567,761 (mean ± SD) reads per sample. After quality control and removal of host contamination, 1,850,381,554 high-quality reads were generated, with 115,648,847 ± 35,201,440 reads per sample. A total of 11,249,270 contigs were generated by the *de novo* assembly (the N50 length of 1,561 ± 258 bp), with 703,079 ± 221,496 reads for each sample. The rumen metagenome contains 98.39% bacteria, 0.97% eukaryota, 0.59% archaea, and 0.05% viruses. The PCoA plot visually showed the distinct separation of bacteria between CON and DTR based on the Bray-Curtis distance, eukaryota, archaea, and viruses have no significantly change (Figures 1A–D). At the domain level, the relative abundance of bacteria, eukaryota, archaea, and viruses were no significantly less in the hindgut of DTR sheep compared with CON (Figure 2). At the level of microbial phylum, Firmicutes, Bacteroidota, Proteobacteria, Spirochaetota, Cyanobacteria, Verrucomicrobiota, Fibrobacterota, Methanobacteriota, Campylobacterota, and Desulfobacterota, Evosea are the main phylum (Supplementary Figure S1). At the genus level, the dominant microbiota were Cryptobacteroides, followed by Succiniclasticum Alistipes, Faecousia, Phocaeicola, RF16, Treponema, Succinivibrio, HGM04593, HGM20899 and UBA4372 (Supplementary Figure S2).

**Figure 3:**
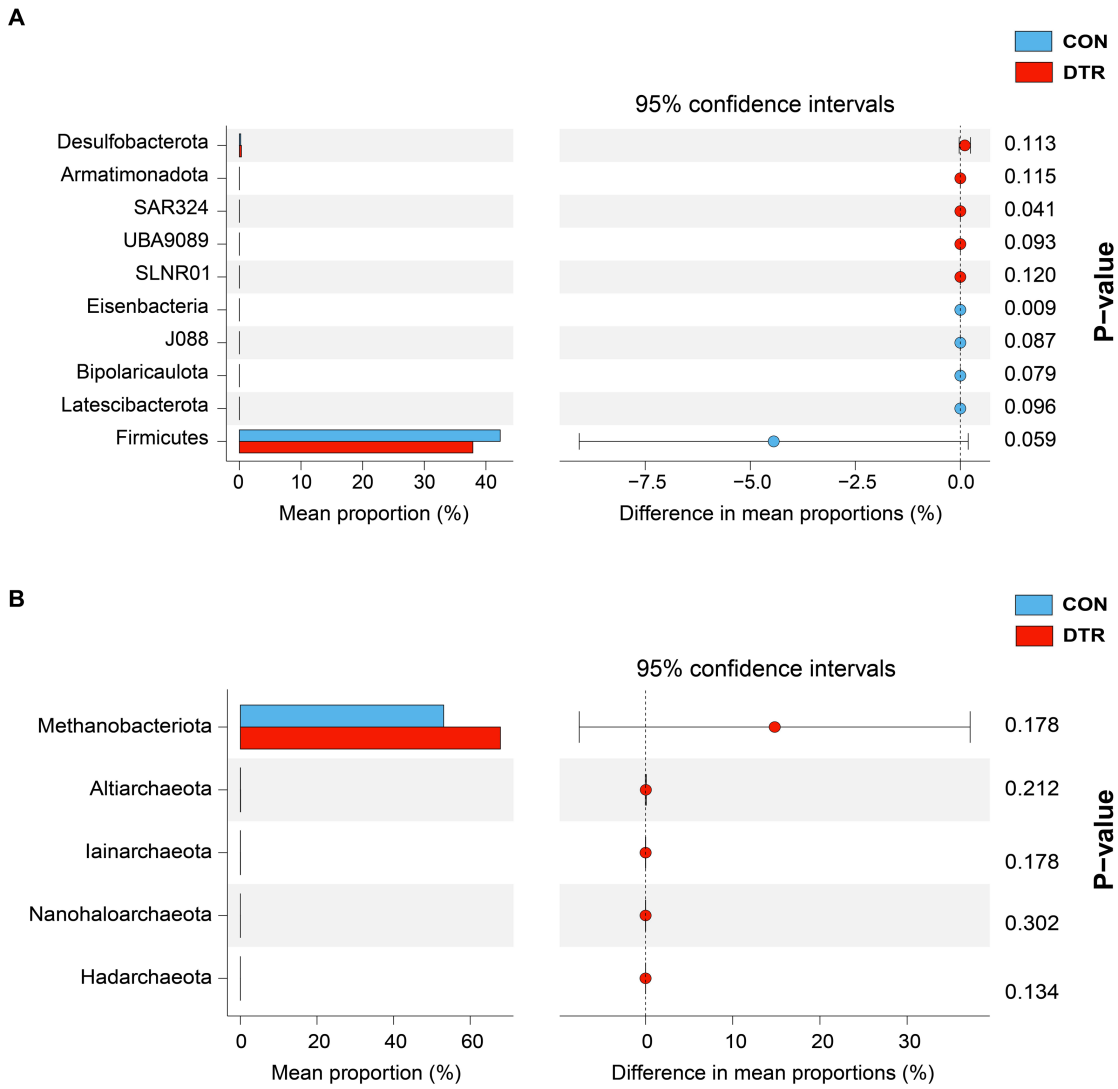
Comparison of the main hindgut microbial taxa at the phylum level between the CON and DTR sheeps based on the Wilcoxon rank-sum test. **(A)** The top 10 phyla within bacteria. **(B)** The top five phyla within archaea.

**Figure 4:**
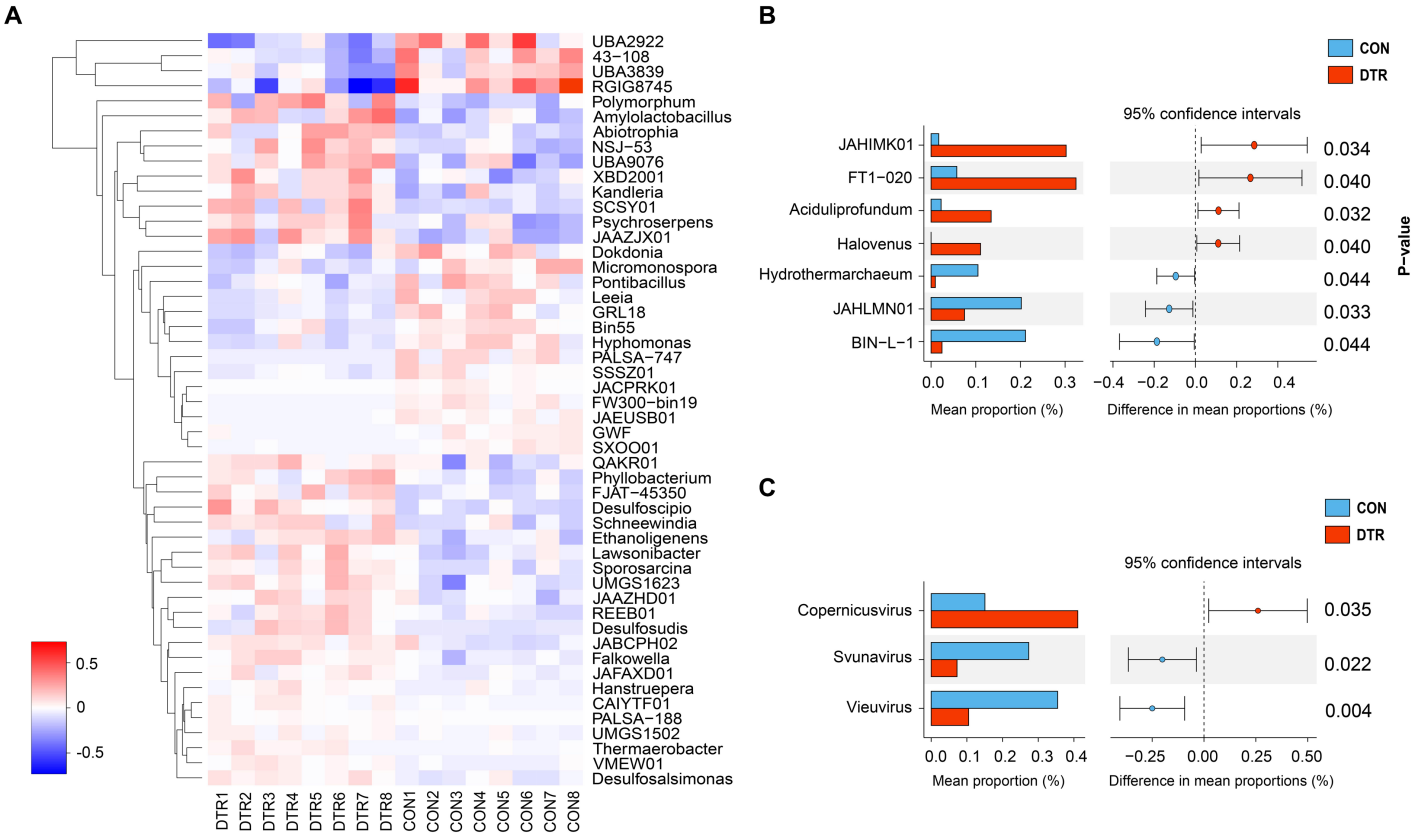
Differential hindgut microbial taxa at the genus level between the CON and DTR sheeps based on the Wilcoxon rank-sum test. **(A)** Heatmap of the top 50 differential genera within bacteria. **(B)** All differential genera within archaea. **(C)** All differential genera within viruses.

The comparison of the hindgut microbial taxa at the phylum and genus levels between the CON and DTR groups was focused on bacteria and archaea. The analytical results of the top 10 bacterial phyla obtained by the Wilcoxon rank-sum test are shown in Figure 2. The phyla BSAR324 exhibited higher abundances (*p* = 0.041) in the hindgut of the DTR sheeps (Figure 3A). No differences were observed in the top five phyla within archaea between two groups (Figure 3B). The top 50 differential bacterial genera are shown in Figure 4A. The relative abundances of 51 genera including Polymorphum, Amylolactobacillus, Abiotrophia, Phyllobacterium, Desulfoscipio, Schneewindia, Ethanoligenens, Lawsonibacter, and Sporosarcina were greater (*p* < 0.05) in the DTR sheeps, whereas the relative abundances of nine genera, including UBA2922, 43-108, UBA3839, and RGIG8745 were greater (*p* < 0.05) in the CON sheep. The archaea genera of JAHIMK01, FT1-020, Aciduliprofundum, and Halovenus showed a high abundance (*p* < 0.05) in the DTR sheep, whereas the genera Hydrothermarchaeum, JAHLMNO1, and BIN-L-1 were low abundant (*p* < 0.05) in the CON sheep (Figure 4B). The virus genera of Svunavirus and Vieuvirus showed a low abundance (*p* < 0.05) in the DTR sheep, whereas the genera Copernicusvirus was more abundant (*p* < 0.05) in the DTR sheep compared with the CON sheep (Figure 4C).

**Figure 5:**
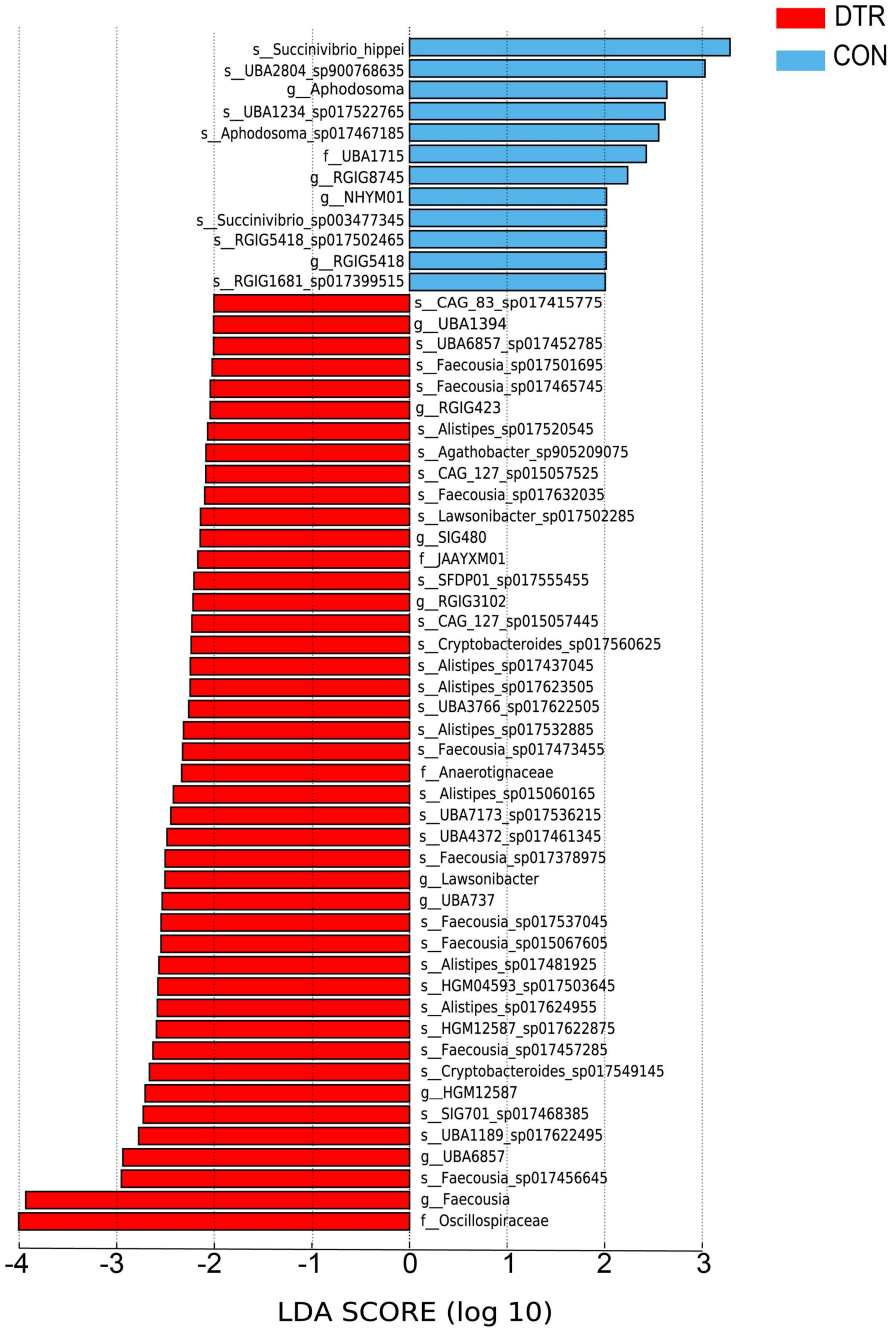
Differential rumen microbial taxa at the different levels between the CON and DTR sheep based on LEfSe.

Comparisons of the taxa at the different levels were performed using LEfSe with the non-parametric factorial Kruskal–Wallis and Wilcoxon rank-sum tests. A total of seven species, four genes and one phylum were more abundant in the CON sheep compared to the DTR sheep (LDA > 2.5 and *p* < 0.05; Figure 5), whereas 32 species, 9 genes and 3 phyla were significantly enriched in the CON sheep (LDA > 2.5 and *p* < 0.05; Figure 5).

### Functional analysis of the microbiome

**Figure 6:**
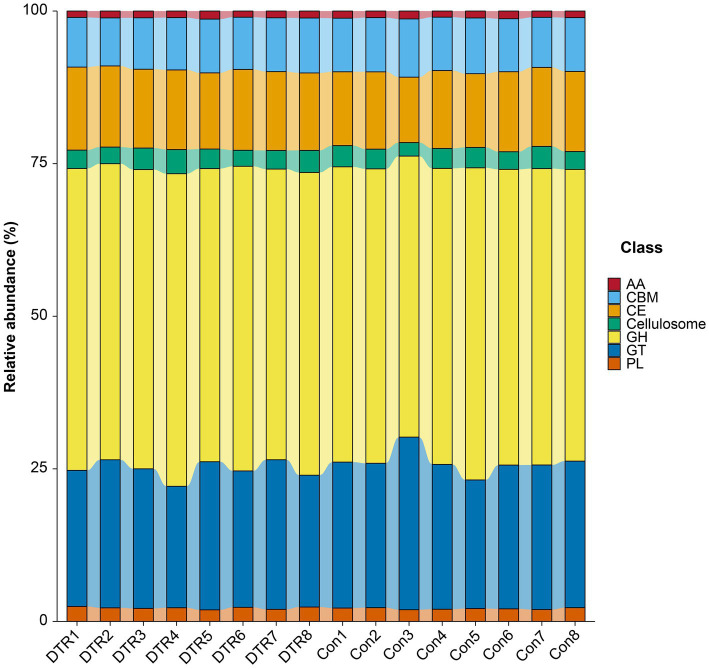
Cazy composition diagram at class level. Glycoside hydrolases (GH), glycosyltransferases (GT), carbohydrate esterases (CE), carbohydrate-binding modules (CBM), polysaccharide lyases (PL), and exhibited auxiliary activities (AA).

Due to the fact that carbohydrates are degraded by multiple enzymes, we focused on the differences in the profiles of CAZymes between the CON and DTR sheep. As shown in Figure 6, the CAZymes community consisted of glycoside hydrolases (GH; 48.85%), glycosyltransferases (GT; 23.26%), carbohydrate esterases (CE; 12.77%), carbohydrate-binding modules (CBM; 8.67%), polysaccharide lyases (PL; 2.16%), Cellulosome (3.19%), and exhibited auxiliary activities (AA; 1.12%). At the class level, there is no significant variation of CAZy between the CON and DTR groups (Supplementary Figure S3), and PCoA also shows no apparent separation at the Class level (Supplementary Figure S4). Among the CAZymes that participated in degrading carbohydrates, three families (2 of CBMs, and 1 of GT) were enriched in the CON sheep, whereas 13 families (4 of CBMs, 7 of GHs and 2 of PLs) were enriched in the CON cows (Supplementary Table 2).

**Figure 7:**
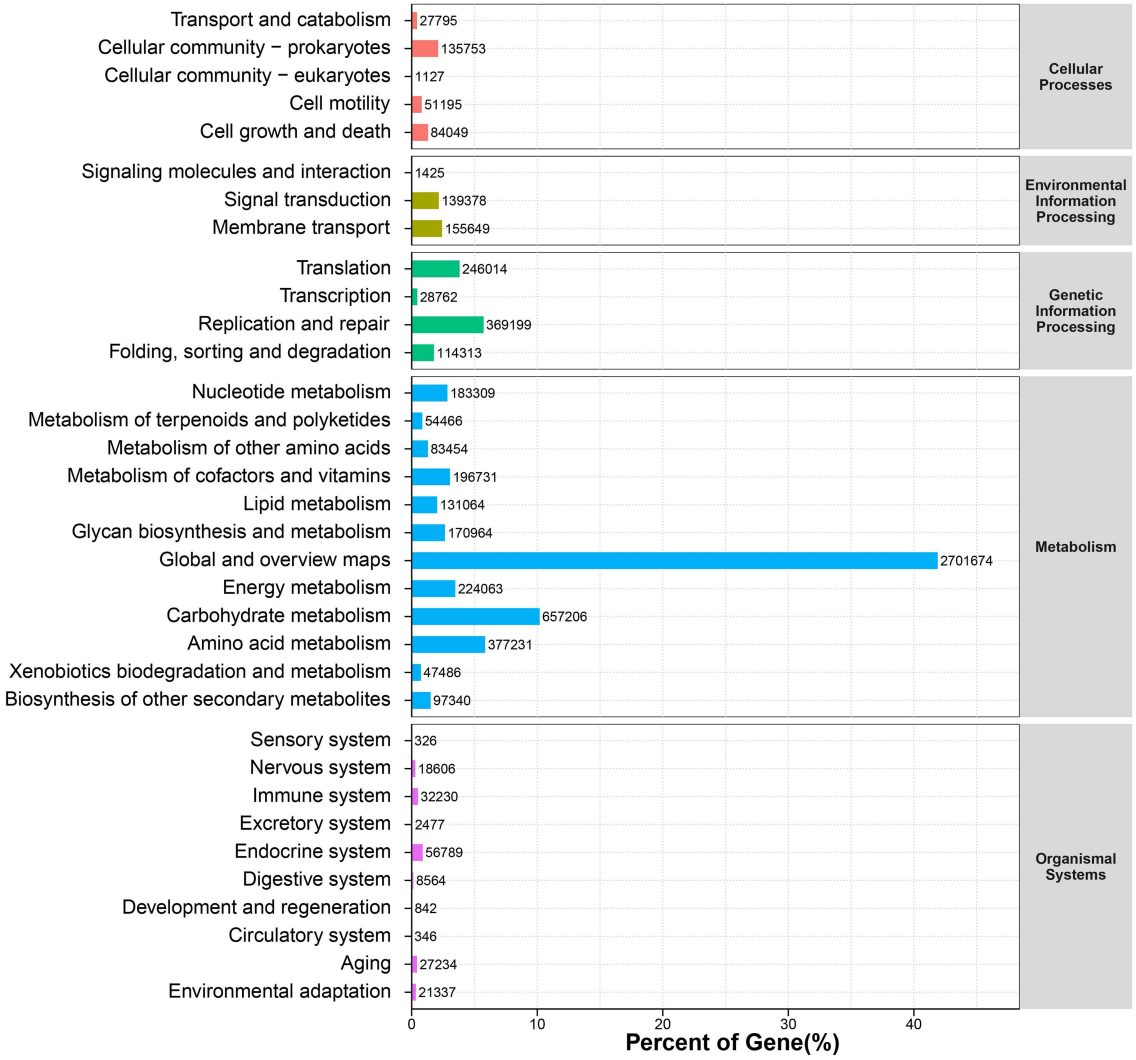
Functional features profiling at KEGG pathway.

Figure 7 displays the main functions of KEGG in 16 sheep, which primarily include five pathways: Cellular Processes, Environmental Information Processing, Genetic Information Processing, Metabolism, and Organismal Systems. The PCoA plot based on the Bray-Curtis distance showed a clear separation of two groups at pathway level 2 (Supplementary Figure S5). We considered 348 endogenous third-level metabolic pathways as hindgut microbial pathways in the KEGG profiles for the further analysis. As shown in Supplementary Table 3, compared to the CON group, there were 31 upregulated metabolic pathways (including methane metabolism, propanoate metabolism, microbial metabolism in diverse environments, purine metabolism, carbon metabolism, starch and sucrose metabolism, pentose phosphate pathway, and glycerolipid metabolism, among others, *p* < 0.05) and 23 downregulated metabolic pathways (including Dorso-ventral axis formation, bile secretion, sphingolipid signaling pathway, growth hormone synthesis, secretion and action, cGMP-PKG signaling pathway, fcepsilon RI signaling pathway, and calcium signaling pathway, among others, *p* < 0.05).

## Discussion

Under normal circumstances, adding food or industrial by-products to an animal’s diet may not significantly affect the animal’s performance. This could be because the content of various active or nutritional components in the by-products does not reach concentrations that have a noticeable effect (Sarker et al., 2022; Khurana et al., 2023). Previous studies have indicated that the use of tea and its by-products in monogastric animals can significantly improve their production performance, such as increasing egg production rate, egg weight, and other factors (Wang et al., 2018; Chen et al., 2023). However, in this study, no significant impact of DTR on sheep’s production performance was observed, which may be attributed to the unique rumen fermentation characteristic of ruminant animals. Kolling et al. (2018) used green tea extract as an additive in dairy cow production and found that it did not affect the production performance of dairy cows. However, it promoted the health and rumen fermentation pattern of the cows. However, in Acharya et al.’s (2020) research report, it was indicated that adding green tea extract could increase milk production in periparturient cows. This may be attributed to the specific physiological state of periparturient cows, and the enhanced production performance of these cows after adding green tea extract might be due to the potent antioxidant effects of green tea. In conclusion, the application of green tea by-products as a feed resource or feed additive in sheep’s diet will not have negative effects on sheep’s production.

Green tea is rich in various active compounds, which are high-quality immune modulators. These active compounds can stimulate the activation of macrophages and B cells, promoting the formation of antibodies (Ding et al., 2018). The active compounds in tea, such as catechins and EGCG, can modulate the activity of immune cells, promoting the activation of macrophages and lymphocytes, and enhancing their proliferation and secretion of immunoglobulins (Yahfoufi et al., 2018). Immunoglobulins are antibody-active animal proteins secreted by plasma cells and play a crucial role in both specific and non-specific immunity. In this experiment, DTR significantly increased the concentrations of IgA, IgG, and IgM in sheep, indicating that DTR exerted a significant immunomodulatory effect in sheep. This finding is consistent with the results of Yuan et al.’s (2023) study. In production, oxidative stress is considered a major factor leading to animal diseases. Green tea and its by-products have been shown to enhance the antioxidant status in animals (Lu et al., 2014). For example, in dairy cows (Ma et al., 2021) and laying hens (Ling et al., 2022), the polyphenolic compounds in green tea can scavenge various oxygen free radicals, including superoxide anion, singlet oxygen, peroxynitrite, and hypochlorous acid (Severino et al., 2009). They can also achieve antioxidant effects by reducing the expression of redox-sensitive transcription factors such as NF-κB and activator protein-1, inhibiting the activity of “pro-oxidant” enzymes, and increasing the activity of antioxidant enzymes such as GSH-Px (Frei and Higdon, 2003). In this study, the addition of DTR significantly increased the concentrations of T-SOD, GSH-Px, and CAT, indicating that DTR has significant antioxidant activity in sheep. This finding is similar to the research results of Ma et al. (2021). This may be attributed to the abundant polyphenols and EGCG content in DTR. However, the specific mechanism by which DTR exerts antioxidant activity in sheep needs further investigation. ALB and HDL are important “regulatory” and “transport” functional indicators in animal blood. They play crucial roles in the metabolism of substances like glycerol. The decrease in ALB and HDL after the addition of DTR may be due to the enhanced metabolism of lipid substances in sheep, which is also related to the strengthened Glycerolipid metabolism pathway observed in the experiment. The significant decrease in TG supports this observation. The changes in these indicators are also significantly correlated with the enhancement of sheep’s immunity and antioxidant capacity. However, the specific mechanisms have not been studied yet.

In this study, the consumption of DTR by sheep had the greatest impact on bacteria, with the most pronounced changes observed at the phylum level for SAR324 and Eisenbacteria. Among them, SAR324 is a widely distributed bacterial group on earth, and its metabolic characteristics are mainly reflected in genes that encode a novel particulate hydrocarbon monooxygenase (pHMO), degradation pathways for corresponding alcohols and short-chain fatty acids, dissimilatory sulfur oxidation, formate dehydrogenase (FDH), and nitrite reductase (NirK). It is primarily associated with lithotrophy, heterotrophy, and alkane oxidation, among other metabolic functions (Sheik et al., 2014; Boeuf et al., 2021). This also explains one of the reasons why the addition of DTR leads to an increase in methane metabolism pathways. However, there is limited knowledge about Eisenbacteria and their potential involvement in carbohydrate metabolism and methane metabolism (Poghosyan et al., 2020). At the bacterial genus level, we observed a significant increase in the abundance of bacteria such as Ammylolactobacillus, Phyllobacterium, Ethanoligenens, Lawsonibacter, Staphylococcus, Desulfosudis, etc., after adding DTR. This increase may be related to lactate fermentation (Zheng et al., 2020), lipid metabolism (Zamlynska et al., 2017), hydrogen and ethanol production fermentation (Li et al., 2019), butyric acid production (Sakamoto et al., 2018), and immune metabolism (Vaskevicius et al., 2023). However, the specific functions associated with the significantly decreased bacterial genera at the genus level have not been reported yet. At the level of archaea and viruses, the specific effects of DTR on microbial changes are yet to be further explored. However, overall, DTR does not have a negative impact on the microbial structure in the sheep’s hindgut. This is consistent with previously reported research findings (Ramdani et al., 2013).

The degradation of carbohydrates by gut microbiota requires various enzymes, including GH, PL, CE, GT, AA, Cellulosome, and CBM. The addition of DTR significantly affects the distribution of various CAZy in the gut, with most of these changes belonging to the GH family, which are polysaccharide-degrading enzymes produced by fiber-degrading bacteria. The addition of DTR significantly affects the distribution of various CAZy in the gut, with most of these changes belonging to the GH, which are polysaccharide-degrading enzymes produced by fiber-degrading bacteria (Flint et al., 2012). The increase in GH abundance may be due to changes in bacterial composition. For instance, the GH1 family plays a crucial role in carbohydrate degradation within organisms, breaking down complex polysaccharides into simpler sugar molecules, and participating in the degradation of cellulose, galactosides, polysaccharides, and oxalates to provide energy metabolism and other biological processes (Cota et al., 2015; Strazzulli et al., 2019). GH3 is involved in the degradation of β-glucoside substrates and drug metabolism. Regarding GH, GH25, GH27, GH112, GH120, and GH154 enzymes show higher abundance after the addition of DTR, indicating that supplementing DTR can enhance microbial digestion and absorption of food. Similarly, the changes of CBM (Arai et al., 2003) and PL (Yang et al., 2021) are also related to fiber digestion and starch digestion. In general, the addition of DTR enhances the ability of fiber digestion in the hindgut.

With the changes in the hindgut microbial structure, there are differences in the KEGG functional profiles between the CON group and the DTR group. The addition of DTR significantly enhances pathways such as methane metabolism, propanoate metabolism, purine metabolism, starch and sucrose metabolism, and glycerolipid metabolism. The enhancement of methane metabolism may be related to the improved fiber digestion, as mentioned earlier in the changes in the abundance of fiber-digesting enzymes (Li et al., 2022). The alterations in propanoate metabolism, purine metabolism, and glycerolipid metabolism pathways also indicate the effectiveness of DTR in enhancing carbohydrate metabolism (Hong et al., 2022). The changes in glycerolipid metabolism may also be related to the significant decrease in TG observed in this study. After the addition of DTR, the sphingolipid signaling pathway significantly decreases, which may be related to DTR’s regulation of animal lipid metabolism and alteration of the animal’s gastrointestinal microbiota (Yuan et al., 2023).

## Conclusion

This study provides new insights into the application of DTR in sheep production. Supplementing DTR significantly improves the sheep’s immune and antioxidant indicators and promotes their fiber digestion capability. This is of great significance to the feeding system for sheep. DTR is a promising natural additive that has positive effects on animal health and the environment. Further exploration of DTR’s effects on rumen fermentation, microbial communities, and fiber metabolism will contribute to its further development and application in ruminant animals.

## Data availability statement

The datasets presented in this study can be found in online repositories. The names of the repository/repositories and accession number(s) can be found in the article/Supplementary material.

## Ethics statement

The animal study was approved by Animal Care and Use Committee at Nanjing Agricultural University. The study was conducted in accordance with the local legislation and institutional requirements.

## Author contributions

LG: Writing – original draft, Writing – review & editing. SY: Writing – original draft, Data curation, Formal analysis. FC: Writing – original draft, Data curation, Project administration, Software. KZ: Writing – original draft, Methodology, Formal analysis, Investigation, Software. ML: Writing – original draft, Data curation, Methodology, Supervision, Conceptualization, Resources. ZP: Writing – original draft, Project administration, Investigation, Visualization. XS: Writing – original draft, Data curation, Supervision, Conceptualization, Investigation. LL: Writing – review & editing.

## Footnotes

^1^https://ensembl.org/index.html

^2^https://github.com/hyattpd/Prodigal

^3^https://ftp.ncbi.nlm.nih.gov/blast/db/FASTA/

^4^http://www.cazy.org/
