# Supplementary material for: Dried tea residue can alter the blood metabolism and the composition and functionality of the intestinal microbiota in Hu sheep
Source: Front Microbiol. 2023 Nov 3;14:1289743. doi: 10.3389/fmicb.2023.1289743 (PMC10655126; doi:10.3389/fmicb.2023.1289743)
Supplement: Supplementary file 1 [file Data_Sheet_1.ZIP › supplement/Supplementary material.docx]

Supplementary table 1. Composition and nutrient levels of the basal diet (dry matter basis %).

| Ingredients | | Nutrient levels^2^ | |
| --- | --- | --- | --- |
| Corn / % | 20.40 | ME/(MJ/kg) | 10.14 |
| Wheat bran / % | 3.56 | CP/% | 14.09 |
| Soybean meal / % | 12.80 | EE/% | 5.06 |
| Corn silage / % | 10.00 | NDF/% | 50.30 |
| Peanut straw / % | 50.00 | ADF/% | 22.4 |
| NaCl / % | 0.28 | Ca/% | 1.09 |
| NaCO_3_ / % | 0.64 | P/% | 0.43 |
| CaCO_3_ / % | 0.24 |  |  |
| CaHCO_3_ / % | 0.08 |  |  |
| Premix^1^ / % | 2.00 |  |  |
| Toyal / % | 100.00 |  |  |

ME = metabolic energy; CP = crude protein; EE = ether extract, NDF = neutral detergent fiber; ADF = acid detergent fiber.

^1^The premix provided the following per kilogram of the diet: Cu 16.0 mg, Fe 35.0 m, Mn 30.0 mg, Zn 80.0 mg, I 0.5 mg, Se 0.10 mg, Co 0.03 mg, VA 14400 IU, VD 4 400 IU, VE 30 mg.

^2^The metabolic energy was calculated and the rest was measured.


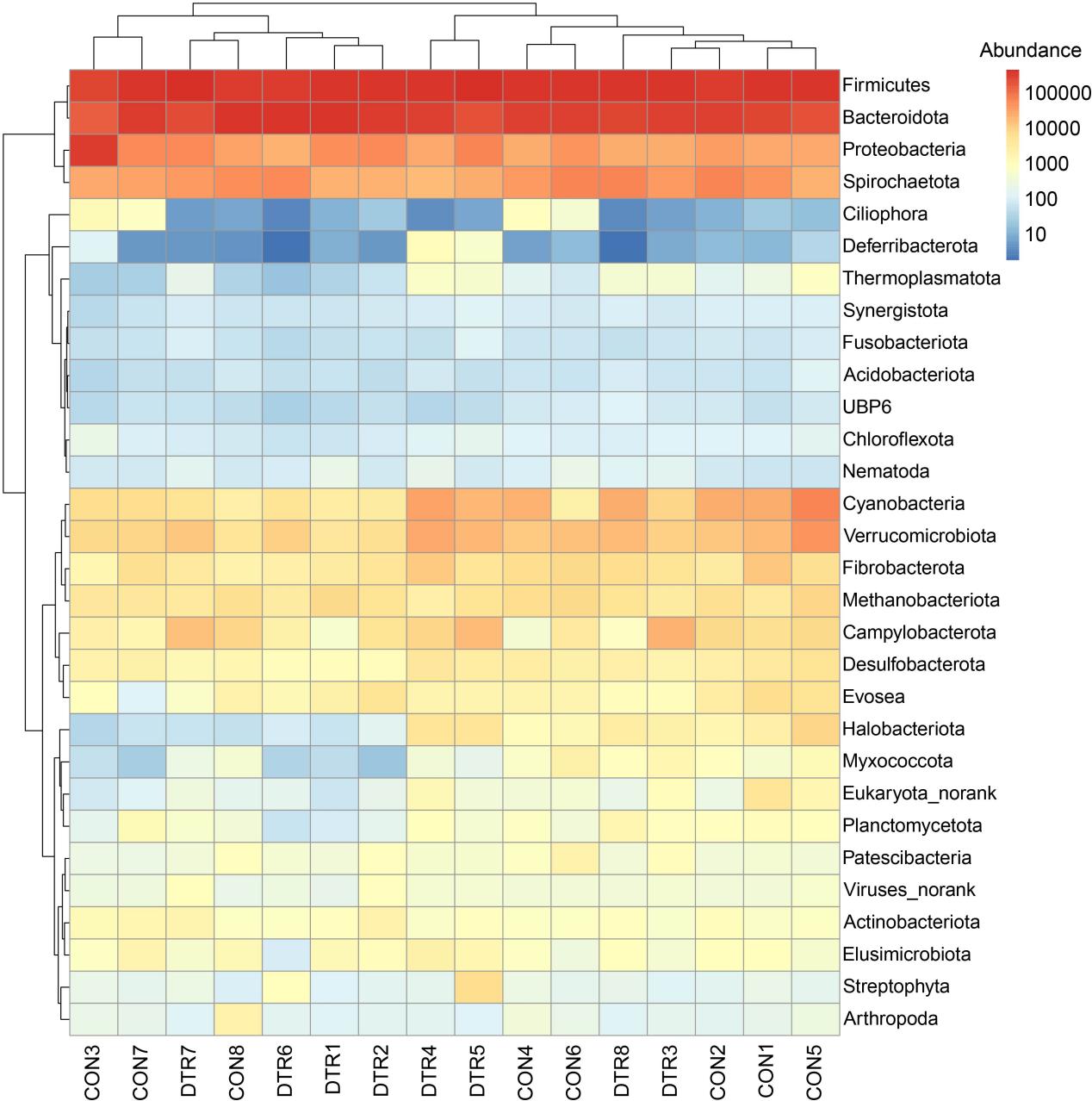
Fig. S1 The predominant microbial composition chart of all samples at the phylum level


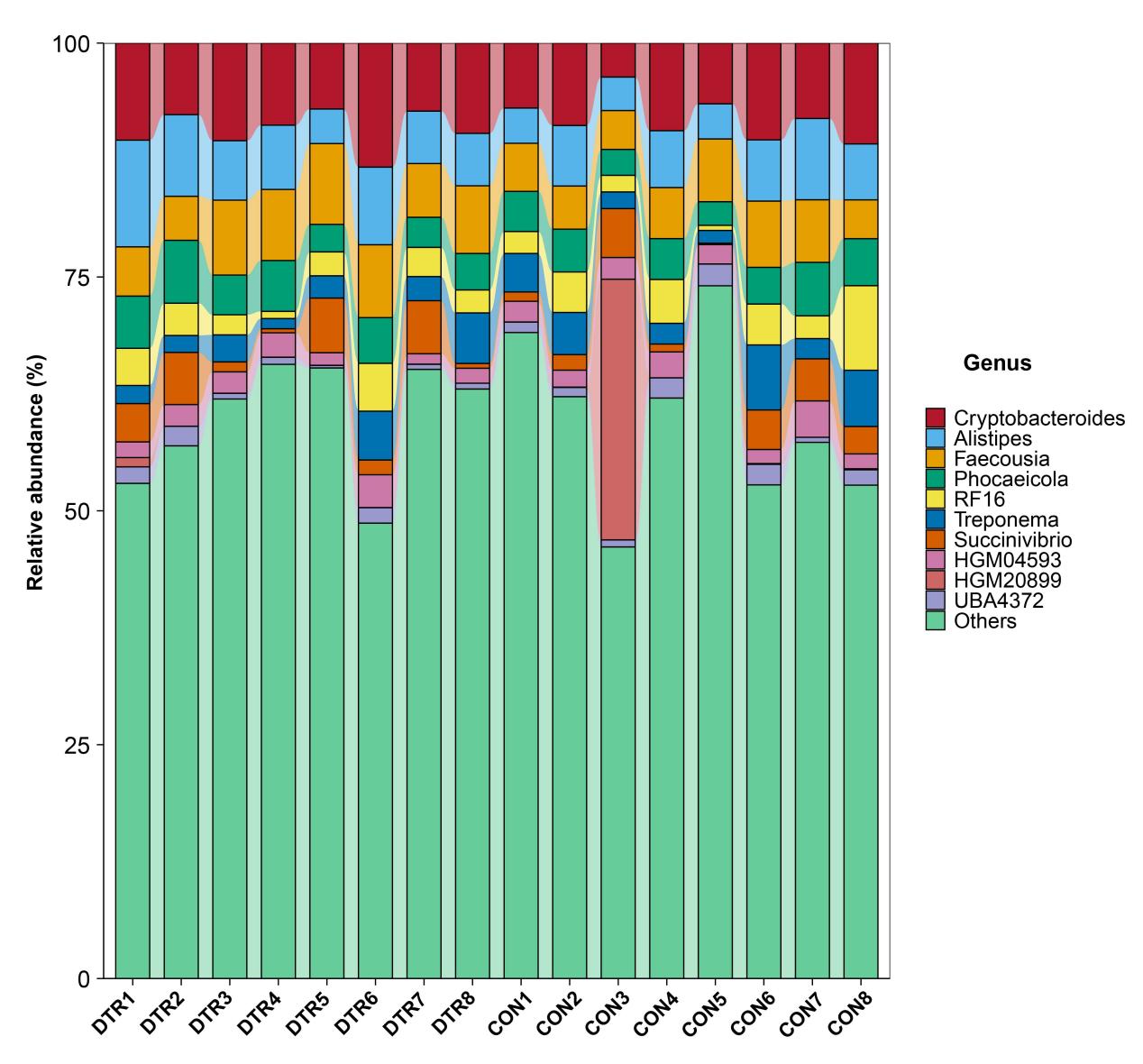


Fig. S2 The predominant microbial composition chart of all samples at the gene level


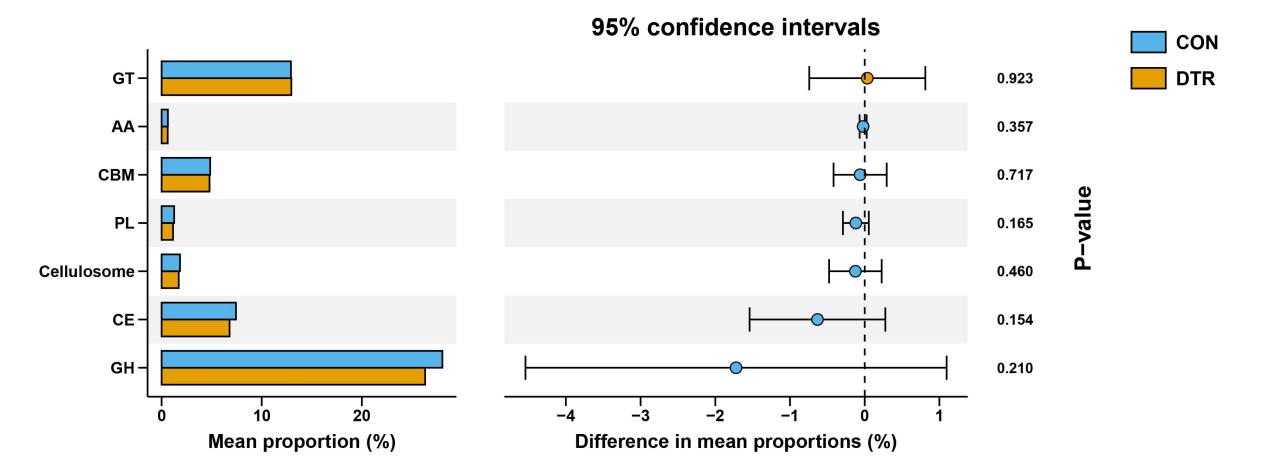


Fig. S3 Differential analysis of Cazy at the Class level. glycoside hydrolases (GH), glycosyltransferases (GT), carbohydrate esterases (CE), carbohydrate-binding modules (CBM), polysaccharide lyases (PL), and exhibited auxiliary activities (AA).


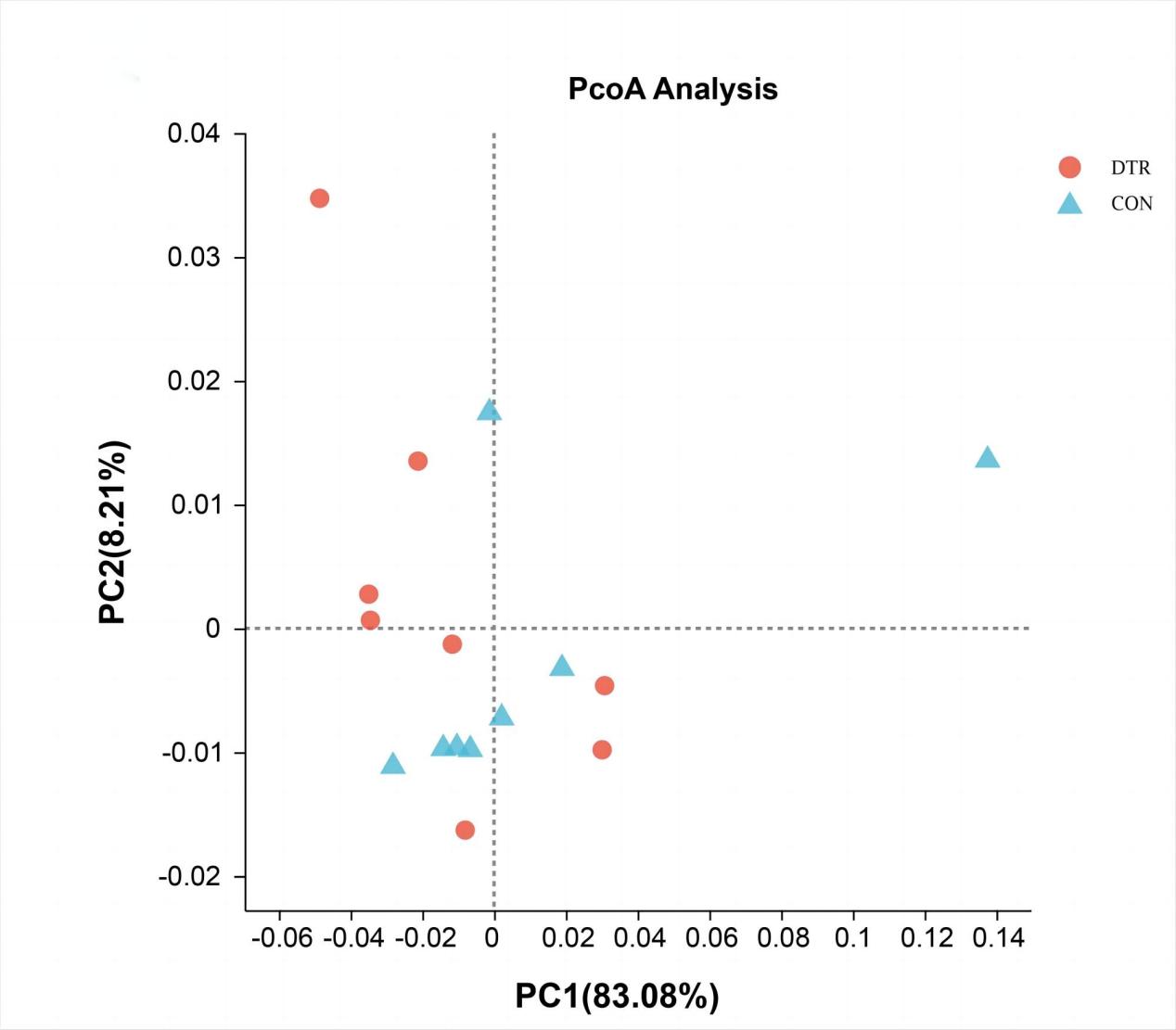


Fig. S4 PcoA analysis of Cazy at the Class level.


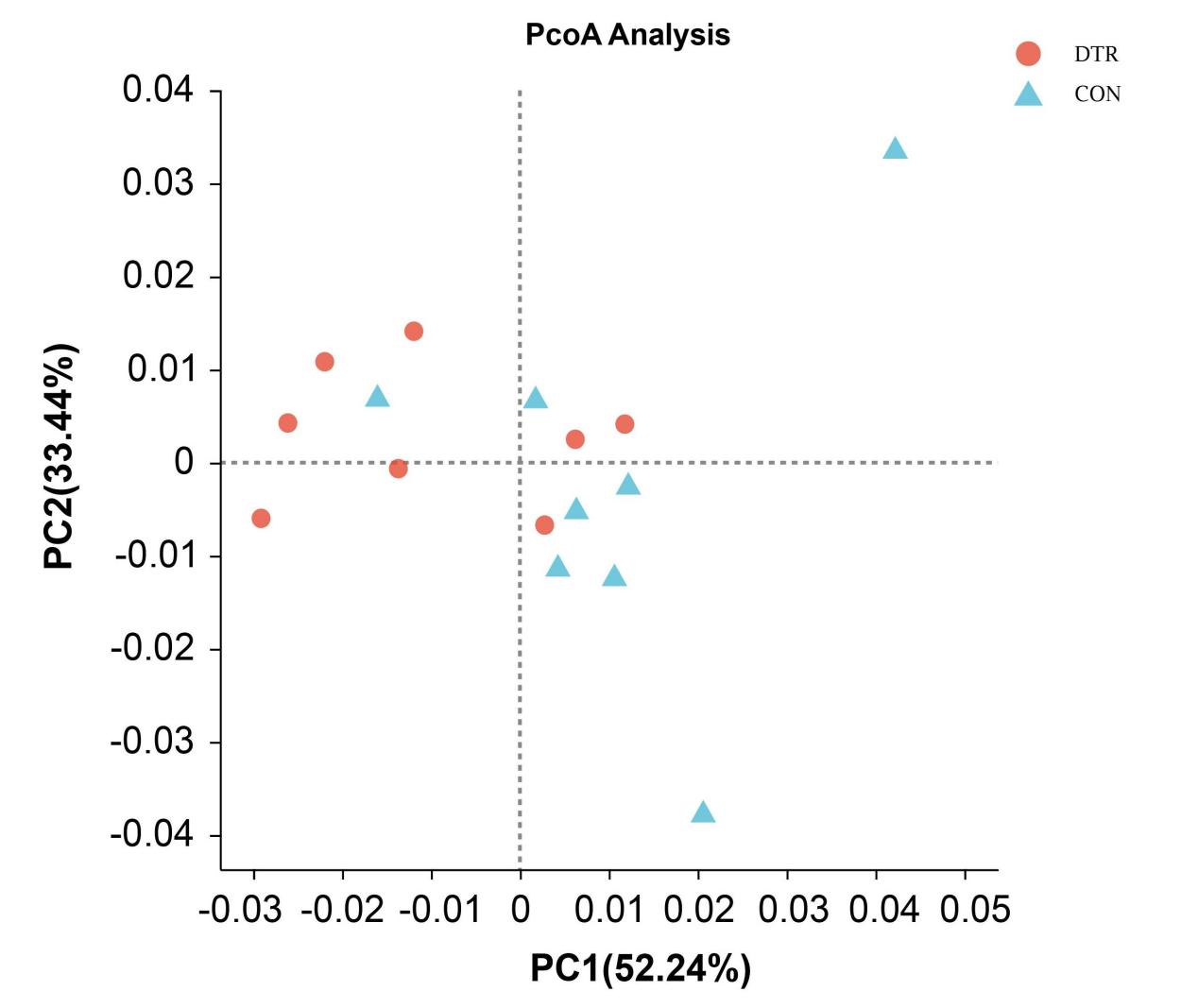


Fig. S5 PcoA analysis of Cazy at the KEGG level2.
